# Sex/Gender Differences in the Cognitive‐Aging Effects of Head Injuries due to Interpersonal Violence

**DOI:** 10.1002/alz.091609

**Published:** 2025-01-09

**Authors:** Douglas William Hanes

**Affiliations:** ^1^ Renaissance School of Medicine at Stony Brook University, Stony Brook, NY, USA; Dalla Lana School of Public Health, University of Toronto, Toronto, ON Canada

## Abstract

**Background:**

Traumatic brain injury is a risk factor for worse later‐life brain health, including dementia. Yet the role of interpersonal violence and its gendered nature in the TBI–cognition relationship has yet to be fully studied. While men and women alike commit and experience violence, gender‐based violence (GBV)—which primarily targets women, transgender and gender‐nonconforming people, and from which they tend to suffer worse injuries than men—is understudied. Using population‐based survey data, we examine gender differences in cognitive effects of self‐reported head injuries due to interpersonal violence.

**Method:**

We use data from the Health and Retirement Study (HRS; n = ), a survey of Americans age 51+. It gathered self‐reported lifetime head‐injury data in 2014, allowing participants to specify injury causes; we restricted our analysis to participants injured in a physical altercation or shot in the head. Participants also indicated injury severity in several binary variables. We calculated summary statistics to compare injury prevalence between men and women. To assess cognitive effects, we used multilevel regressions with subsequent cognitive performance (27 points possible) as the outcome variable and interaction between injury severity and sex/gender as the primary predictor variable, adjusting for race/ethnicity, education, and wealth.

**Result:**

While men were likelier to report head injuries due to fights (c^2^ = 12.884; p < 0.001), women were over twice as likely to report ongoing health problems due to head injury (c^2^ = 10.638; p = 0.001), likelier to have been hospitalized due to a fight or gunshot‐caused head injury (c^2^ = 5.533; p = 0.019), and nearly 5x likelier to report losing consciousness due to choking (c^2^ = 5.069; p = 0.024). Women hospitalized due to a fight or gunshot experienced worse cognitive effects (b = ‐2.428; p = 0.004) than hospitalized men relative to unhospitalized men (b = ‐2.600, p = 0.005 and b = ‐3.372, p < 0.001, respectively).

**Conclusion:**

In keeping with existing literature, women suffer worse injuries than men from interpersonal violence; women also have worse cognitive harms due to head injuries than do men. The reasons for these outcomes, and their relationship to GBV, requires further investigation.

